# Heart Block Initiated by Candlenut Ingestion

**DOI:** 10.1155/2022/3679968

**Published:** 2022-05-30

**Authors:** Osayi Lawani, Mark Winter

**Affiliations:** ^1^University of Houston College of Medicine/HCA Houston Healthcare, USA; ^2^Southeast Texas Poison Center, University of Texas Medical Branch, USA

## Abstract

The candlenut tree is a tropical plant that has a vast number of uses which include fertilizer, dye, ink for tattooing, and fuel. The inner seed of the nut is the most utilized portion of the plant and is often sold as a food additive, natural laxative, or a weight loss supplement. Unfortunately, the seed itself is very toxic when consumed whole and in its raw state. Typical symptoms of toxicity include abdominal pain, vomiting, and diarrhea. Rarely, it can cause cardiac dysrhythmias, most commonly bradycardia and atrioventricular heart block. We present a case of a young adult female with no significant past medical history who developed typical symptoms of toxicity, as well as atrioventricular heart block following ingestion of a candlenut. Most documented cases describe complete resolution of gastrointestinal and cardiac symptoms about one week following ingestion; however, treatment while inpatient can consist of inotropes or vasopressor support, intravenous fluid hydration, electrolyte replacement, and antiemetics. Although the mechanism of action remains unclear, this report provides physicians with an understanding of the risks of ingestion and the knowledge of typical management of the toxic effects of the candlenut.

## 1. Introduction

*Aleurites moluccana*, otherwise known as the kukui nut or candlenut tree, is a medium-sized tree that is distinctive by its silver-green leaves, and originates from the Indonesian-Malaysian region of Southeast Asia [1]. The plant is now widely found throughout the tropics which includes Hawaii, the Caribbean, and South America [1]. It has a vast number of uses which may depend on its overall local need and cultural significance. Almost every part of the tree (leaves, fruit, and seeds) can be repurposed. Examples of some of its uses include fuel, dye, ink for tattooing, waterproofing, and fertilizer, and it can be found in some traditional Asian medicines [2].

Many of the candlenut tree's uses are found from its nut or the inner seed [1]. The candlenut is typically edible in small quantities when cooked; however, it is generally very toxic when ingested raw [1]. Although the popularity of the nut lies with its use as a weight loss supplement or laxative, symptoms of reported toxicity are commonly abdominal pain, vomiting, and diarrhea [2]. A rare complication that has been seen in those who have eaten a whole raw nut has been cardiac dysrhythmias. The most frequently seen arrhythmias reported were bradycardia and atrioventricular heart block [1]. Due to a chance incidence of morbidity and possible mortality, several countries have banned its use [3]. In this report, we present a case of a young female who was found to have these rare effects shortly following the ingestion of a candlenut.

## 2. Case History

A 21-year-old female with no significant past medical history presented to the emergency department with complaints of nausea and vomiting. The vomitus was nonbloody and nonbilious, and she stated that she experienced five intermittent episodes for about two hours before coming to the emergency department. Laboratory tests were taken, and results were all within normal range. The patient was administered antiemetics and one liter of intravenous fluids which improved her symptoms, and she was consequently discharged home. The following day, the patient returned to the emergency department with generalized malaise and continued nausea and vomiting that increased in frequency to about every 30 minutes. Additionally, the patient noted a decrease in her heart rate per her smart watch and a new sensation of numbness and tingling in her extremities while she was vomiting.

**Figure 1:**
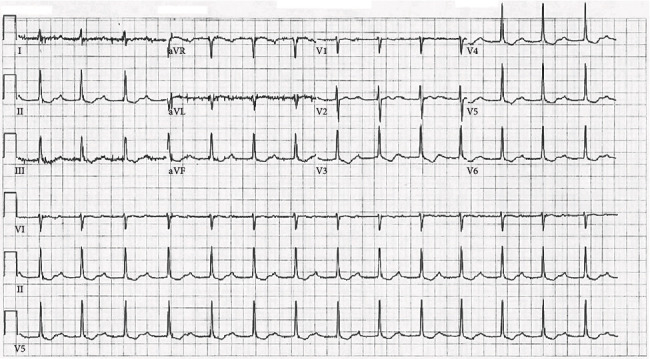
An electrocardiogram showing first-degree atrioventricular heart block two days following ingestion of a candlenut. Also noted is a characteristic scooping of the ST segment known as the *digitalis effect*.

Vital signs were stable upon arrival. The physical exam was negative for any abnormalities. Laboratory results were again unremarkable. Urinalysis was negative for any illicit drug use. A chest X-ray was also unremarkable. An electrocardiogram (ECG) was performed and showed a first-degree atrioventricular (AV) heart block with a PR interval of 0.354 seconds and a heart rate of 86 beats per minute (Figure 1). The patient stated that the only recent change in her diet was the ingestion of a candlenut the day of her first emergency room visit, which she had obtained from her uncle who had recently returned from Latin America. She intended to take the nut as a weight loss supplement and ingested a whole nut with one can of an energy drink. About 45 minutes afterward she began experiencing nausea, vomiting, and abdominal pain. Poison control was contacted and it was suggested that the patient may have toxicity similar to what is seen with yellow oleander (*Thevetia peruviana*) poisoning. As per poison control, the patient was empirically administered three vials of digoxin immune Fab, one ampule of bicarbonate to increase her venous pH from 7.40 to 7.45, and started on 20% fat emulsion intravenously. The patient was later admitted to the intensive care unit (ICU) for further evaluation.

**Figure 2:**
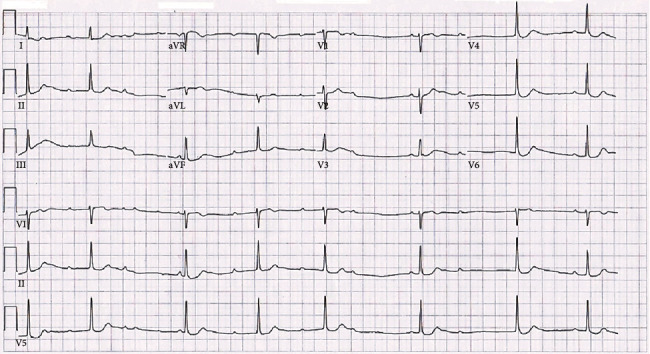
An electrocardiogram showing third-degree atrioventricular heart block three days following ingestion of a candlenut.

A peripherally inserted central catheter line was placed in the right arm, and the patient was continued on the infusion of 20% fat emulsion per poison control recommendations. The following day, the patient began to have complaints of dizziness with ambulation, and an abnormal rhythm was noticed on telemetry. A second ECG was performed and noted a new third-degree AV heart block with a ventricular rate of 45 beats per minute (Figure 2). Poison control recommended placement of a pacemaker. The patient was placed on intravenous dopamine, and cardiology was consulted for further evaluation.

It was advised by the poison center that the patient's potassium be kept greater than 4 mmol/L and magnesium greater than 2 mg/dL. A transthoracic echocardiogram was performed and showed no abnormalities. Per poison control recommendations, the fat emulsion therapy was stopped on day three of admission. On day four, the patient had reported improvement in her nausea and vomiting and was also able to be weaned off dopamine as her high-grade heart block resolved. However, she continued to display first-degree heart block with a heart rate of about 60 to 70 beats per minute. On day five, the patient was transferred from the ICU to a telemetry unit for further care and monitoring of her electrolytes. Ultimately, the patient showed marked improvement over the next two days with resolution of her first-degree heart block to normal sinus rhythm; she was then cleared by cardiology and the primary care team for discharge. The patient was instructed to refrain from ingesting candlenuts for weight loss or any other homeopathic use.

## 3. Discussion

Candlenuts are commonly marketed as a food additive, natural laxative, or a weight loss supplement [3]. Currently, the exact mechanism for its function within the body that leads to toxicity is unknown. The toxic component of the tree lies within the inner most part of the nut, the seed [3]. One known compound contained within the seed is phorbol esters, a chemical that produces a potent inflammatory response [4]. The gastrointestinal and cardiac systems are particularly affected by phorbol as it activates protein kinase C (PKC), which irreversibly inserts itself into the cell membrane of these two organ systems [4]. For the heart, phorbol can produce a negative inotropic effect on the myocytes, depress cell contractility by decreasing sarcolemma calcium influx, and also reduces the number of beta-adrenoceptors and their affinity for beta-agonists [1]. Additionally, activation of PKC can lead to phosphorylation and thus activation of calcium channels that can affect the function of the heart [5]. This could explain why patients may experience arrhythmias after ingestion of an undercooked candlenut.

Reports of cardiac dysfunction following the ingestion of the candlenut are rare; however, when cardiac abnormalities have been documented, they have included bradycardia, first-degree AV heart block, and second-degree AV heart block types 1 and 2 [6]. There are several causes of this disruption of impulse conduction within the heart, which include drug toxicity, hyperkalemia, ischemia, structural heart issues, and excessive vagal stimulation. Differential diagnoses include organophosphate poisoning, autoimmune disease in younger patients, and herbal medication overdose [1]. The abnormal electrical activity can be transient or permanent and can occur within the AV conduction system or originate from dysfunction below the system [6].

With first-degree AV block, the impulse is slowed to where the PR interval is greater than 300 milliseconds and the P-wave may be buried underneath the preceding T-wave [6]. This is a benign condition which usually does not require any treatment. With second-degree Mobitz type 1 AV heart block, the PR interval displays progressive prolongation with every beat until a nonconducted QRS occurs after a P-wave. This rhythm is also typically benign, and asymptomatic patients do not require treatment [6]. Symptomatic individuals will respond to atropine dosing, and, in rare instances, permanent pacemaker placement may be necessary [6]. Higher degrees of AV block have severe dysfunction of the conduction system and typically require urgent hospital admission and a permanent pacemaker [6].

One phenomenon that is also linked to candlenut ingestion is the change in ECG wave morphology. In a few reports, it was found that the ECG of those who consumed the seed displayed a characteristic scooping of the ST segment known as the *digitalis effect*, which is usually seen in patients taking the antiarrhythmic drug digoxin [7]. This was especially noted in adolescents and young adults that had ingested the seed as they were less likely to have taken digoxin [7]. The patient in this case was noted to have these effects and also did not take digoxin (Figures 1 and 2). This may be a result of the potential increased amount of available calcium in the cardiac myocytes from phorbol's activation of PKC, as candlenuts have not been positively identified as a substance that contains cardiac glycosides. In a few cases, some patients were also noted to have elevated serum digoxin levels after candlenut ingestion, and each patient denied taking or being prescribed digoxin [2, 3]. It is unknown, however, if exposure to candlenuts could cause a falsely elevated serum digoxin level [3]. One paper suggests that possibly some individuals with arrhythmias that were thought to have eaten a candlenut may have mistakenly ingested a yellow oleander seed, which does contain cardiac glycosides [8]. This could be true in some instances; however, in this case, the patient was initially administered three vials of digoxin immune Fab which had no overall effect, and the patient eventually progressed to third-degree AV heart block a day later. Further studies will likely need to be performed to assess this unusual cardiac occurrence.

Although there was no specific sampling done to provide supportive evidence of candlenut ingestion other than the patients' subjective report, there was a temporal association between the reported ingestion and the related gastrointestinal and cardiac signs and symptoms that the patient experienced. The toxicity of this nut has not gone unnoticed. Similar to the ban placed on the herbal supplement ma huang (Ephedra species) by the United States in 2004 due to adverse cardiovascular events, the candlenut has also been banned in several countries, including Argentina, Spain, and Brazil [3, 9]. Unfortunately, the nut continues to be available for sale by small vendors and on select websites found on the Internet [2].

## 4. Conclusion

The candlenut has been advertised as a weight loss supplement and is sold worldwide [2]. Currently, there are only a limited number of documented cases of candlenut toxicity in humans, especially those leading to cardiovascular events. The recurring theme with all noted cases is that the cardiotoxic effects of AV heart block or bradycardia, as well as gastrointestinal disturbances, resolve on average of about six to seven days after nut ingestion. Treatment involves symptomatic management, which can comprise of inotropes or vasopressor support, intravenous fluid hydration, electrolyte replacement, and antiemetics [1]. Charcoal and gastric lavage may also be indicated in some cases [1]. Although the mechanism of action of the candlenuts' effects remains unclear, this report can provide physicians with an understanding of the risks of ingestion and the knowledge of typical management of the toxic effects of the candlenut.

## Data Availability

Data is available upon request to the corresponding author.
